# Insight into anaerobic methanotrophy from ^13^C/^12^C- amino acids and ^14^C/^12^C-ANME cells in seafloor microbial ecology

**DOI:** 10.1038/s41598-018-31004-5

**Published:** 2018-09-24

**Authors:** Yoshinori Takano, Yoshito Chikaraishi, Hiroyuki Imachi, Yosuke Miyairi, Nanako O. Ogawa, Masanori Kaneko, Yusuke Yokoyama, Martin Krüger, Naohiko Ohkouchi

**Affiliations:** 1 Department of Biogeochemistry, Japan Agency for Marine-Earth Science and Technology (JAMSTEC), 2-15 Natsushima, Yokosuka, 237-0061 Japan; 2 Research and Development Center for Marine Resources, Japan Agency for Marine-Earth Science and Technology (JAMSTEC), 2-15 Natsushima, Yokosuka, 237-0061 Japan; 3 Institute of Low Temperature Science, Faculty of Environmental Earth Science, Hokkaido University, N19W8 Kita-ku, Sapporo, 060-0819 Japan; 4 Department of Subsurface Geobiological Analysis and Research, Japan Agency for Marine-Earth Science and Technology (JAMSTEC), 2-15 Natsushima, Yokosuka, 237-0061 Japan; 5 Atmosphere and Ocean Research Institute, University of Tokyo, 5-1-5 Kashiwanoha, Kashiwa, 277-8564 Japan; 6 Research Institute for Geo-Resources and Environment, National Institute of Advanced Industrial Science and Technology (AIST), Central 7, 1-1-1 Higashi, Tsukuba, 305-8567 Japan; 7 Federal Institute for Geosciences and Natural Resources (BGR), Stilleweg 2, D-30655 Hannover, Germany

## Abstract

Oceanic methane from global deep-sea sediment is largely consumed through microbially mediated sulfate-coupled oxidation, resulting in ^13^C-depleted cell biomass of anaerobic methanotrophic archaea (ANME). The general ecological importance of subseafloor ANME has been well recognized in the last two decades. However, the crucial biochemical pathways for the overall anaerobic oxidation of methane (AOM) still remain enigmatic. Here, methanotrophic pathways were analyzed to trace ^13^C-depleted amino acid biosynthesis in two clades of ANME (ANME-1 and ANME-2) from the Black Sea. Compound-specific analysis of ANME-dominated microbial mats showed a significant ^13^C-depletion trend in association with increasing carbon numbers in protein-derived amino acid families (*e.g.*, the pyruvate family in the order of alanine, valine, isoleucine and leucine was down to −114‰). This result indicates a stepwise elongation of ^13^C-depleted carbon during amino acid biosynthesis. The overall results suggest that intracellular protein amino acids and the most ^13^C-depleted signature of leucine, which has a specific branched-chain structure, are potentially propagated as isoprenoid precursor molecules into archaeal biosynthesis, resulting in the extremely ^13^C- and ^14^C-depleted nature of ANME cells in the deep microbial oasis.

## Introduction

**Figure 1:**
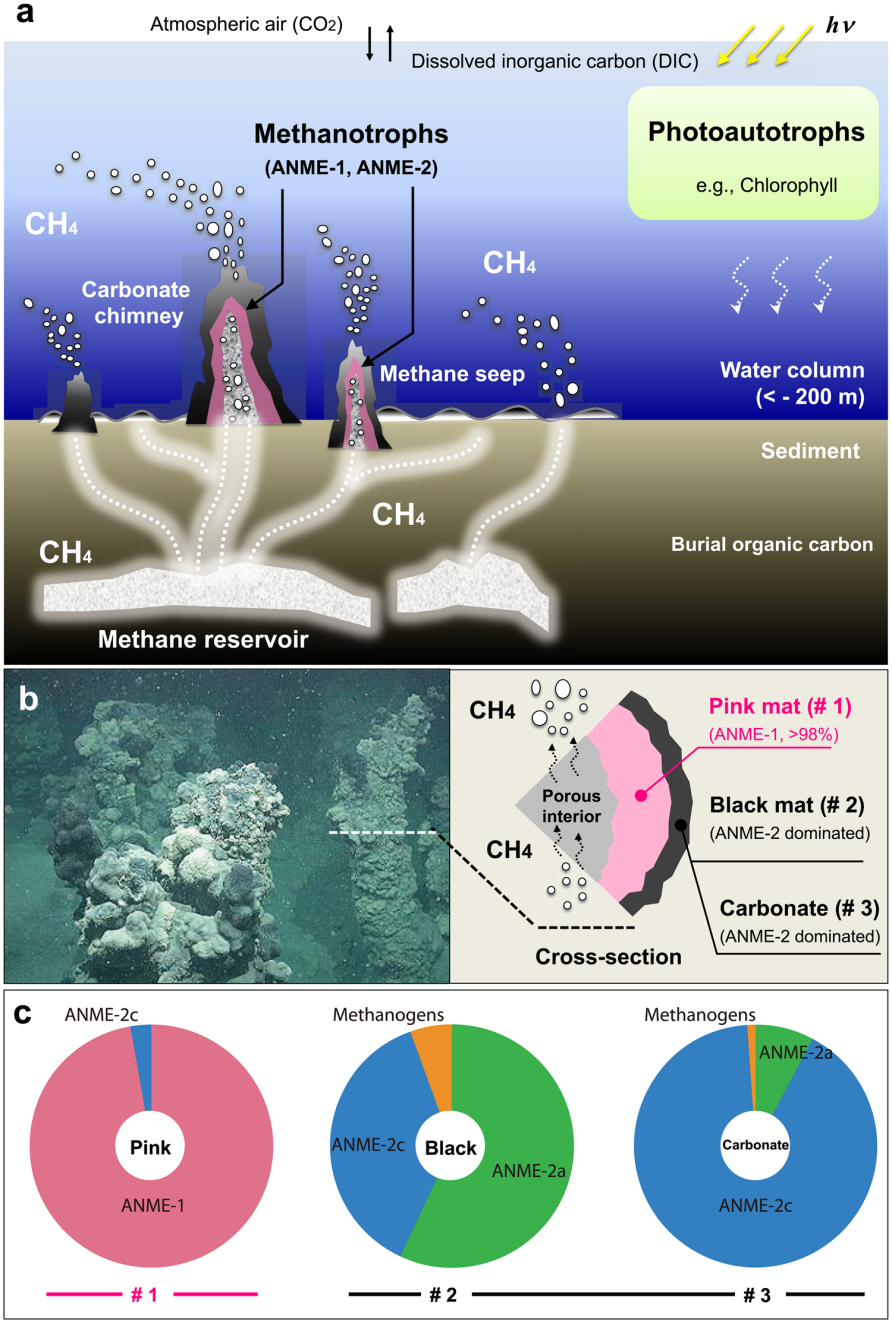
ANME-dominated microbial mats from the Black Sea. (**a**) Benthic methane seep environment of the Black Sea. The earliest description of the Black Sea mats and carbonate chimneys was reported by Luth and coworkers^44^. Please see the Supplementary Movie S1 for methane venting from carbonate chimneys. (**b**) Interior and exterior sections of chimney structure habitat location (image capture during the expedition) showing a pink mat (ANME-1-dominated), black mat, and carbonate precipitate (ANME-2-dominated)^30^. Photo credit: *R/V Meteor* cruise M72/1 science party (taken by M. Krüger). In the northwestern Black Sea at the Ukrainian shelf and slope area, a number of active gas seeps (at least 2778 sites)^45^ occur along the shelf edge near the Crimean Peninsula^46–52^. (**c**) The community structures of the ANME samples were determined by methyl coenzyme M reductase A (*mcrA*) gene-based clone analyses of the pink mat, black mat, and carbonate samples. ANME-1 and ANME-2 dominate the pink and black sections, respectively. Highly pure ANME-1 was observed in the pink section (Supplementary Fig. S4), whereas there was some diversity in the black and carbonate samples (Supplementary Figs S5 and S6). Based on the lipid analysis of ANME-1, the relative abundance of archaeal lipids was >98% (Supplementary Fig. S4). The isotopic composition of carbon (δ^13^C, ‰ vs. VPDB) and nitrogen (δ^15^N, ‰ vs. Air) and the radiocarbon (Δ^14^C, ‰) data for bulk ANME cell biomass are shown in Fig. 4.

Microorganisms play a central role in both methane production and consumption in the global carbon cycle. The anaerobic oxidation of methane (AOM) is an important microbial process that controls the release of greenhouse gas from oceanic sediment^1^. Since the discovery of extremely ^13^C-depleted lipids produced by modern anaerobic methanotrophic archaea (ANME) in deep-sea sediments^2–4^, we have recognized the ^13^C-depleted isotopic signatures (*ca*. −110‰) as an ongoing AOM process from biogeochemical models for lipid biomarker records^5,6^, even in hydrothermally active seafloor settings^7,8^. Three phylogenetic groups of anaerobic methanotrophic archaea (ANME) have currently been identified, namely, ANME-1 (with subgroups a and b), ANME-2 (with subgroups a, b, c, and d), and ANME-3, which mediate AOM *via* sulfate (CH_4_ + SO_4_^2−^ → HCO_3_^−^ + HS^−^ + H_2_O)^9^, nitrate^10^, iron, and manganese^11^. AOM requires methyl coenzyme M reductase^12,13^, which catalyzes anaerobic methanotrophy through reverse methanogenesis^14–16^. Because obtaining pure cultures in the laboratory and isolating ANME are difficult, the biochemical mechanisms that control the AOM process, especially the pathways leading to ^13^C-depleted cell biomass, remain largely unknown. To better define the AOM process by focusing on biogeochemistry, we investigated the carbon isotopic composition of amino acids, the fundamental building blocks of proteins, in ANME-1- and ANME-2-dominated mats collected from the northwestern Black Sea (Fig. 1). ANME-1 and ANME-2 are observed in tall reef-like chimney structures (up to *ca*. 5 m height, 1 m diameter) composed of carbonates and dense microbial biomass (<10^10^ cells cm^−3^) (ref.^17^) where the methane seep rises vertically through the porous calcified interior. Therefore, we conducted compound-specific carbon isotope (^13^C/^12^C) analysis of 10 individual amino acids in the form of *N*-pivaloyl isopropyl ester derivatives and archaeal isoprenoid lipids together with radiocarbon (^14^C/^12^C) analysis of ANME cell using an accelerator mass spectrometry.

### The carbon isotope chemistry of ANME-dominated microbial mats

**Figure 2:**
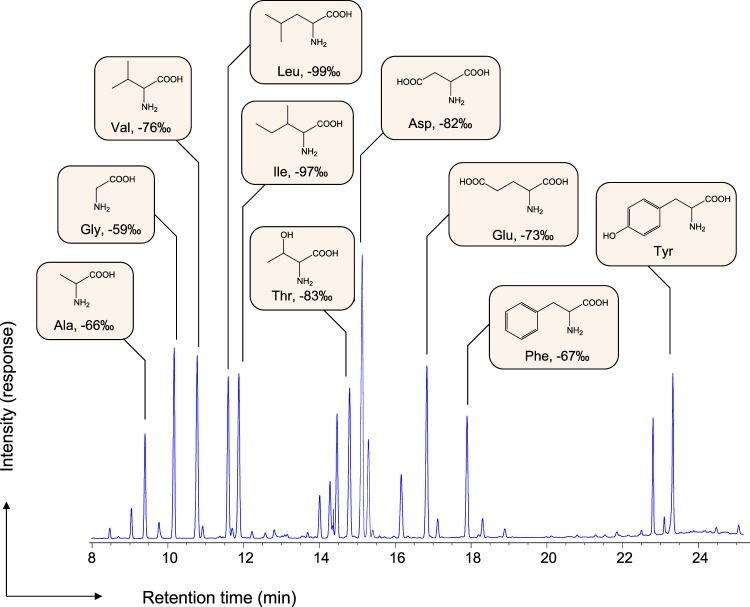
Gas chromatographic separation of ^13^C-depleted amino acids from ANME-1. Please see the analytical accuracy in Supplementary Fig. S3 and Table S1. Abbreviations: Ala, alanine (underivatized formula, C_3_H_7_NO_2_); Gly, glycine (C_2_H_5_NO_2_); Val, valine (C_5_H_11_NO_2_); Leu, leucine (C_6_H_13_NO_2_); Ile, isoleucine (C_6_H_13_NO_2_); Thr, threonine (C_4_H_9_NO_3_); Asp, aspartic acid (C_4_H_7_NO_4_) and asparagine after hydrolysis; Glu, glutamic acid (C_5_H_9_NO_4_) and glutamine after hydrolysis; Phe, phenylalanine (C_9_H_11_NO_2_); Tyr, tyrosine (C_9_H_11_NO_3_).

**Figure 3:**
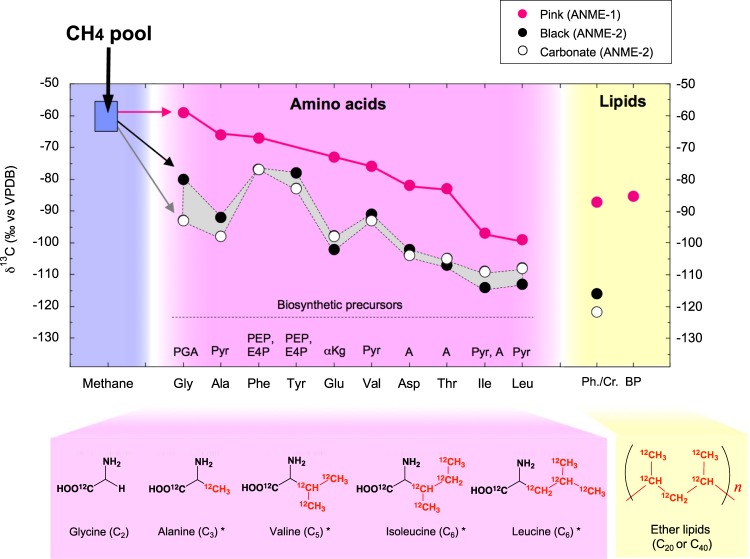
δ^13^C values of amino acids and lipids extracted from ANME mats in the Black Sea. Chemical structure and ^13^C-depletion of neutral amino acids glycine, alanine, valine, isoleucine, and leucine with carbon numbers (C_*n*_) up to C_6_. The asterisks (*) represent pyruvate amino acid family members. Abbreviations: PGA, phosphoglyceric acid; Pyr, pyruvate; A, aspartic acid; αkg, α-ketoglutarate; PEP + E4P, phosphoenolpyruvate + erythrose-4-phosphate. The carbon isotopic composition of the ANME-2-dominated black mat and carbonate included the major archaeal C_20_ isoprenoid (<−116‰, vs. VPDB) (Supplementary Table S1).

Mats from this region provide ideal natural enrichment for the study of methane biogeochemistry and microbial anaerobic methanotrophy mediated by modern ANME communities (Supplementary Fig. S1)^18–21^. To date, the AOM rate in the microbial reefs of the Black Sea is the fastest observed, with an estimated range of 10^3^–10^4^ nmol cm^−3^ day^−1^ (cf. Supplementary Fig. S2)^17,21^. We observed substantial ^13^C depletion in the amino acids from the ANME samples (Fig. 2), as low as to −114‰ relative to the Vienna Pee Dee Belemnite (VPDB) international standard (Fig. 3). Assuming the carbon isotopic composition of the substrate methane in the same area to be −50 to −65‰^21,22^, the isotopic fractionation by the ANME communities associated with amino acid biosynthesis was estimated to be 50–60‰. Methanotrophy by ANME-1 reportedly includes the formation of functionalized one-carbon (C_1_) compounds, such as methanol, methylamine, and methyl sulfide, from initial methane uptake^16^. Dual stable isotope probing (D- & ^13^C-) experiments also suggested that autotrophic carbon fixation from dissolved inorganic carbon (C_1_) occurred in the ANME-1 community^23^. Given that amino acid biosynthetic precursors include *de novo* pathway of pyruvate, phosphoglyceric acid, aspartic acid, α-ketoglutarate, and phosphoenolpyruvate + erythrose-4-phosphate^24^, we expected biosynthetic amino acids with additional ^12^C-carbon elongation to produce more ^13^C-depleted carbon isotopic signatures in ANME communities.

### Branched-chain amino acids in ANME

In the central metabolic pathways of ANME archaea, the pyruvate family includes four major amino acids: alanine, valine, isoleucine, and leucine^16^. Our data showed a stepwise ^13^C-depletion trend associated with the carbon numbers of neutral amino acids containing monoamino and monocarboxylic functional groups (Fig. 3), such as glycine (C_2_), alanine (C_3_), valine (C_5_), isoleucine (C_6_), and leucine (C_6_) (*R*^2^ = 0.91 and *R*^2^ = 0.80 in ANME-1 and ANME-2, respectively). Based on the metagenomic analysis of ANME-1, pyruvate serves as the biosynthetic precursor for L-valine (from 2-keto-isovalerate), L-leucine (2-keto-isocaproate), and L-isoleucine (2-keto-3-methylvaletrate), which are synthesized *de novo* (Fig. 2)^16^. Valine is synthesized by the addition of a ^13^C-depleted acetyl group to the alanine carbon skeleton^24^, resulting in a ^13^C-depleted biosynthetic flow that eventually progresses to leucine. In contrast, chorismate formed *via* the shikimate pathway is a precursor for aromatic amino acids with carbon isotopic compositions comparable to phenylalanine and tyrosine in ANME. The formation of tyrosine by enzymatic hydroxylation is the prephenate dominant metabolic pathway^16^. Of the aspartate amino acid family members, aspartic acid (C_4_; mixed signal with hydrolyzed asparagine) and threonine (C_4_) showed consistent carbon isotopic trends in both the ANME-1 and ANME-2 communities (Fig. 3).

**Figure 4:**
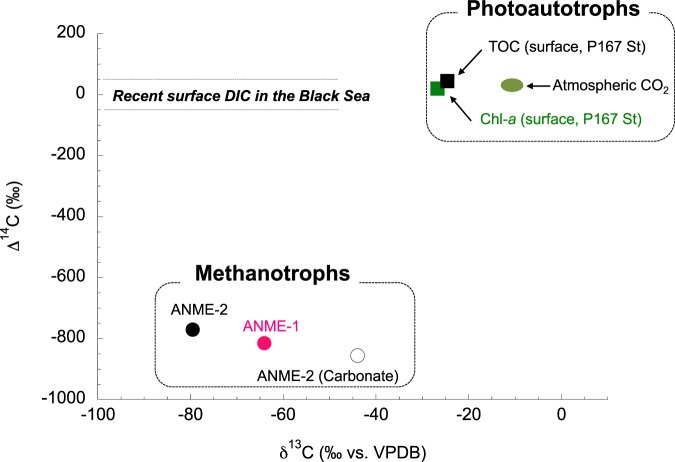
Carbon isotope ratios (^13^C/^12^C and ^14^C/^12^C) between photoautotrophs and methanotrophs in the Black Sea. ANME-specific cross-plot of δ^13^C and Δ^14^C for the present study, compilation of previous reports using surface chlorophyll-*a*, dissolved inorganic carbon (DIC) and surface total organic carbon (TOC) at P167 station^53^, northwestern Black Sea (43°58.88′ N31°30.83′). The same ANME samples (*i.e*., ANME-dominated mats and the carbonate sample discussed in this study) were used in the radiocarbon analysis.

**Figure 5:**
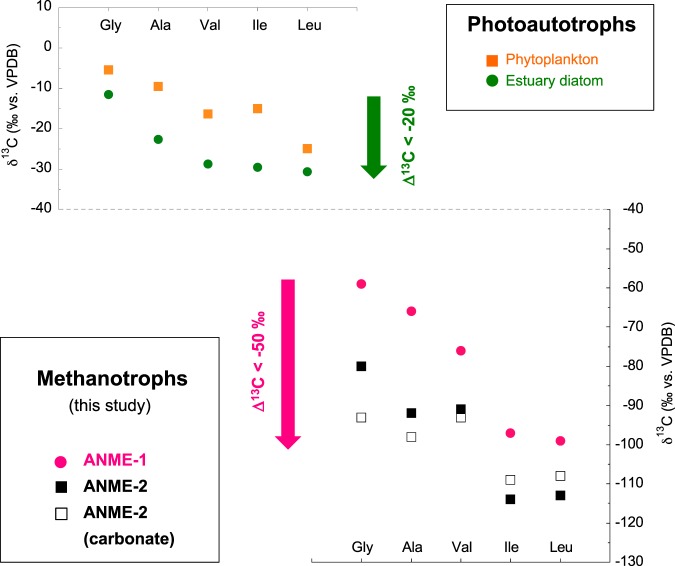
Carbon isotope ratios (^13^C/^12^C) of amino acids. Carbon isotopic composition of neutral amino acids in photoautotrophs (upper diagram: data from ref.^25^) and methanotrophs (lower diagram: this study). ^13^C-depletion proceeds through carbon elongation for C_2_-glycine, C_3_-alanine, C_5_-valine, C_6_-isoleucine, and C_6_-leucine. Δ^13^C is defined as the difference of δ^13^C during these target molecules.

The Δ^14^C values of ANME-1 (−815.3 ± 1.4‰), ANME-2 (−770.4 ± 1.8‰), and carbonate (−855.9 ± 1.6‰) indicated higher ^14^C depletion than in ambient seawater (Fig. 4). The δ^13^C and Δ^14^C cross-plot clearly indicated that venting methane was used directly by ANME cell biomass in the benthic seep chimney. The carbon isotopic order of the present ANME-1 cell biomass was compared with that of oceanic photoautotrophic primary producers because both are end-members in the oceanic carbon cycle (Fig. 5). The carbon isotopic composition of amino acids in representative oceanic photoautotrophs, such as phytoplankton and diatoms^25^, showed a similar trend to those of neutral amino acids (C_2_-glycine >C_3_-alanine >C_5_-valine >C_6_-isoleucine >C_6_-leucine), whereas the ANME communities in this study exhibited a wider carbon isotopic discrimination of the amino acid range than the reference photoautotrophs. Importantly, in both cases, the C_6_-branched-chain amino acids isoleucine and leucine were the most ^13^C-depleted carbon skeletons in the biosynthetic pathway.

### Implications for the fate of ^13^C-depleted amino acids and lipid synthesis in cell biomass

The biosynthesis of isoprenoid lipids from branched-chain amino acids has been postulated^26^ and experimentally verified for *Euryarchaeota* in archaea^27,28^. Therefore, the branched-chain carbon structures of several amino acids have been considered to be important precursors of branched carbon skeletons and branched alkyl lipids. Among the pyruvate amino acid family, leucine is the product most closely related to lipid synthesis, leading to fundamental C_5_-isoprenoid precursors followed by isoprenoid unit elongation. Deuterium (^2^H) probing experiments suggest that partial input from leucine to the mevalonate pathway occurs in archaeal ether lipid synthesis^28^ and bacterial *iso*-C_15_ branched fatty acid synthesis^29^.

The present results and postulated biochemical reaction schemes^28,29^ imply that the fraction (*f*) of intracellular ^13^C-depleted branched-chain amino acids (*i.e*., leucine as *f*_BCAA_) is partially involved in the C_5_ isoprenoid pathway during membrane lipid synthesis, whereas the other fraction of the intracellular acetoacetyl-CoA pathway (*f*_acetyl-CoA_) is substantially involved in this process, as expressed in *f*_acetyl-CoA_ + *f*_BCAA_ = 1 (refs^23,24,26–28^). Alternatively, incorporated extracellular leucine could also produce mevalonate prior to lipid biosynthesis mixotrophically, as suggested by tracer experiments for archaea^28^. Considering physicochemical influences such as environmental factors, archaeal growth phase, and nutrient profiles^27^, the branched-chain amino acid transporter and the permease protein were clearly identified during the metagenomic analysis of ANME-1 sample^16^. Carbon isotopic profiles indicate that leucine and other pyruvate family amino acids potentially play a role in the biosynthesis of the ^13^C-depleted isoprenoid C_5_ precursor. The present ANME lipid analysis supports this interpretation by revealing extremely ^13^C-depleted branched-chain amino acids and isoprenoid lipids (Fig. 3; Supplementary Fig. S4). Similarly, this observation is consistent with previous results showing that ^13^C-depleted ether lipids (e.g., C_20_ and C_40_ isoprenoid units, including hydroxyl ether lipids) are found in ANME layers in the Black Sea^19–21^. Our results advance the biochemical understanding of benthic methane biogeochemistry driven by ANME habitats.

## Methods

### Sampling location of anaerobic methanotrophic archaea

Anaerobic methanotrophic archaea (ANME)−1- and ANME-2-dominated microbial mats and carbonate samples were collected from the northwestern Black Sea during the *R/V Meteor* cruise M72/1 (44°46.46′N, 31°59.50′E, depth 235 m) (ref.^30^). The samples were separated into an ANME-1-dominated pink microbial mat and ANME-2-dominated black mat and carbonate sections^20,22^. To minimize oxygen contamination, all sampling was performed under a nitrogen atmosphere. The pink mats were separated from the black mats with sterile scalpels. Then, the mats were transferred to glass bottles containing filter-sterilized and Black Sea water. The bottles were sealed with butyl rubber stoppers and plastic screw caps and flushed with methane^30^.

### ANME-specific carbon (^13^C/^12^C, ^14^C/^12^C) and nitrogen (^15^N/^14^N) isotope analysis

Dried samples (*ca*. 10–30 µg) of pink (ANME-1 dominated), black (ANME-2 dominated), and carbonate sections were transferred to precleaned tin cups prior to isotopic analysis. Carbon and nitrogen isotopic compositions and total carbon and nitrogen contents were determined by using an isotope ratio-monitoring mass spectrometer (Thermo Finnigan Delta ^Plus^ XP, Thermo Fisher Scientific) connected to an optimized elemental analyzer (Flash EA1112, CE Instruments)^31^. Carbon and nitrogen isotopic compositions were expressed using conventional δ notation as follows.

$${{\rm{\delta }}}^{{\rm{13}}}{\rm{C}}=[{({}^{{\rm{13}}}{\rm{C}}/{}^{{\rm{12}}}{\rm{C}})}_{{\rm{sample}}}/{({}^{{\rm{13}}}{\rm{C}}/{}^{{\rm{12}}}{\rm{C}})}_{{\rm{standard}}}-{\rm{1}}]\times 1000$$

$${{\rm{\delta }}}^{{\rm{15}}}{\rm{N}}=[{({}^{{\rm{15}}}{\rm{N}}/{}^{{\rm{14}}}{\rm{N}})}_{{\rm{sample}}}/{({}^{{\rm{15}}}{\rm{N}}/{}^{{\rm{14}}}{\rm{N}})}_{{\rm{standard}}}-{\rm{1}}]\times {\rm{1000}}$$

The standard deviations for the carbon and nitrogen isotopic compositions were validated within δ^13^C < ± 0.2‰ (*n* = 7) and δ^15^N < ± 0.3‰ (*n* = 7) with the standard reagent BG-T (ref.^32^). ANME samples were also prepared for radiocarbon measurement by single-stage accelerator mass spectrometry at the Atmosphere and Ocean Research Institute, The University of Tokyo^33,34^. The Δ^14^C notation is defined as

$${{\rm{\Delta }}}^{14}{\rm{C}}=({e}^{-\lambda t}-1)\times 1000,\,{\rm{where}}\,\lambda =1/8267\,{\rm{and}}\,t={\rm{year}}\,{\rm{BP}}.$$

Cross-plots of δ^13^C and Δ^14^C are shown in Supplementary Table S1 and Fig. 4.

### Compound-specific isotope analysis (CSIA) of protein amino acids

CSIA of protein amino acids was performed. After acid hydrolysis of the ANME samples with 6 M HCl (110 °C, 12 h), the amino acid fraction was separated into a hydrophilic fraction for derivatization to *N*-pivaloyl *iso*propyl esters^35–38^ and a lipophilic fraction (hexane/dichloromethane, 6:5, v/v) for further lipid analysis. To eliminate organic and inorganic matrix effects and to improve the baseline resolution and accuracy of CSIA, the amino acid fraction was pretreated by cation-exchange column chromatography (AG-50W-X8 resin; 200–400 mesh, Bio-Rad Laboratories; cf. δ^15^N profiles in ref.^38^). Supplementary Fig. S3 shows the validation of the carbon isotopic composition of *N*-pivaloyl *iso*propyl esters of amino acids before and after column chromatography. Carbon isotopic analyses by online gas chromatography/combustion/isotope ratio mass spectrometry (GC/C/IRMS) were performed with an IRMS (Finnigan Delta Plus XP, Thermo Fisher Scientific) combined with a GC (6890 N, Agilent Technologies) with a capillary column (Ultra2, Agilent Technologies; 25 m × 0.32 mm; film thickness, 0.52 μm) in combustion and reduction furnaces^36,37^. The GC heating program was as follows: 3 min at 40 °C, 40–110 °C at a rate of 15 °C min^−1^, 110–150 °C at a rate of 3 °C min^−1^, 150–220 °C at a rate of 6 °C min^−1^, and an isothermal hold at 220 °C for 17.3 min. We occasionally used an HP-INNOWAX column (Agilent Technologies; 30 m × 0.32 mm; film thickness, 0.50 μm), a general purpose dimethylpolysiloxane column (HP-1, Agilent Technologies; 30 m × 0.32 μm; film thickness, 1.0 μm), and a joint column with DB-23 (30 m x 0.32 mm i.d., 0.25 μm, Agilent Technologies) and Ultra2 for threonine (Thr), tyrosine (Tyr), and aspartic acid (Asp), respectively, to improve baseline resolution^37^.

### Compound-specific isotope analysis (CSIA) of archaeal lipids

Archaeal lipids were assessed in the same ANME samples (*i.e*., C_20_ and C_40_ isoprenoids, Supplementary Fig. S4) using an improved method^39^. After separation of the lipid fraction, an ether cleavage treatment^40,41^ was performed with 57 wt % HI (in H_2_O) in a reaction vial with a PTFE-lined cap at 110 °C for 4 h. After the addition of 5 wt % NaCl aqueous solution (5 mL) and *n*-hexane (5 mL), the *n*-hexane fraction was recovered by liquid/liquid extraction (×3). Then, *n*-hexane (5 mL) and PtO_2_ (5 mg) were added to the sample tube, and hydrogenation was performed by gentle H_2_ gas bubbling at room temperature for 30 min. Finally, the *n*-hexane fraction was recovered for the final fraction prior to gas chromatography with flame ionization detection (GC/FID; 6890, Agilent Technologies) and an HP-5 column (Agilent Technologies; 30 m × 0.32 mm i.d., 0.52 μm film thickness). Based on the lipid analysis of the ANME-1 sample, the relative abundance of archaeal lipids (>98%; Supplementary Fig. S4) was greater than that of bacterial alkyl lipids. The key enzymatic process of anaerobic methanotrophy by methyl-coenzyme M reductase (mcr) with coenzyme factor 430 (F430) has been validated previously, together with the CSIA of isolated F430 (ref.^42^). The results from lipids and F430 are consistent with evidence of a nonsyntrophic AOM process driven by ANME alone^43^.

## Electronic supplementary material

Supplementary Information

Supplementary Movie S1
