# Supplementary material for: Insight into anaerobic methanotrophy from 13C/12C- amino acids and 14C/12C-ANME cells in seafloor microbial ecology
Source: Sci Rep. 2018 Sep 24;8:14070. doi: 10.1038/s41598-018-31004-5 (PMC6155224; doi:10.1038/s41598-018-31004-5)
Supplement: Supplementary file 1 — Supplementary Information [file 41598_2018_31004_MOESM1_ESM.pdf]

*Supplementary Information*

# Insight into anaerobic methanotrophy from $^{13}C/^{12}C$- amino acids and $^{14}C/^{12}C$-ANME cells in seafloor microbial ecology

Yoshinori Takano[1,2]*, Yoshito Chikaraishi[1,3], Hiroyuki Imachi[2,4], Yosuke Miyairi[5], Nanako O. Ogawa[1,2], Masanori Kaneko[1,6], Yusuke Yokoyama[1,5], Martin Krüger[7], and Naohiko Ohkouchi[1,2]

[1]Department of Biogeochemistry, Japan Agency for Marine-Earth Science and Technology (JAMSTEC), 2-15 Natsushima, Yokosuka 237-0061, Japan

[2]Research and Development Center for Marine Resources, Japan Agency for Marine-Earth Science and Technology (JAMSTEC), 2-15 Natsushima, Yokosuka 237-0061, Japan

[3]Institute of Low Temperature Science, Faculty of Environmental Earth Science, Hokkaido University, N19W8 Kita-ku, Sapporo 060-0819, Japan

[4]Department of Subsurface Geobiological Analysis and Research, Japan Agency for Marine-Earth Science and Technology (JAMSTEC), 2-15 Natsushima, Yokosuka 237-0061, Japan

[5]Atmosphere and Ocean Research Institute, University of Tokyo, 5-1-5 Kashiwanoha, Kashiwa 277-8564, Japan

[6]Research Institute for Geo-Resources and Environment, National Institute of Advanced Industrial Science and Technology (AIST), Central 7, 1-1-1 Higashi, Tsukuba 305-8567, Japan

[7]Federal Institute for Geosciences and Natural Resources (BGR), Stilleweg 2, D-30655 Hannover, Germany

*Correspondence to: takano (a) jamstec.go.jp

***Scientific Reports***

**doi:10.1038/s41598-018-31004-5**

**Molecular analysis of 16S rRNA and mcrA genes.** DNA extraction, PCR amplification, clone library construction, and sequencing were performed as described previously [54]. For PCR amplification, the primers Arch21f (ref. 55)/Ar912r (ref. 54) and Luton-mcrA (ref. 56) were used for the construction of the archaeal 16S rRNA gene and *mcrA* gene clone libraries, respectively. The PCR conditions were as follows: denaturation at 95 °C for 40 s, annealing at 52 °C for 30 s, and extension at 72 °C for 1 min for archaeal 16S rRNA gene amplification; and denaturation at 95 °C for 40 s, annealing at 50 °C for 30 s, and extension at 72° C for 1 min for *mcrA* gene amplification. To reduce possible bias caused by PCR amplification, PCR products were obtained by minimizing PCR cycle numbers (20–30 cycles at five-cycle intervals). The 16S rRNA gene clonal sequences were classified into each phylotype by using a threshold of 97% sequence identity. Representative phylotypes were subjected to similarity analysis with the BLAST program[57]. The phylogenetic analysis of the 16S rRNA gene sequence was performed as described previously[58]. A deduced McrA amino acid sequence-based phylogenetic tree was constructed by the neighbour-joining method in the ARB program[59] with 130 amino acid positions and the percentage of acceptance of mutations distance correction. The sequences reported in this study were deposited in the GenBank/EMBL/DDBJ database under accession numbers LC069316 to LC069354 (Supplementary Figs. S5&S6). The biosynthesis pathway from the KEGG database[60] and isotope probing verification of representative archaea[28,61], bacteria[30], and eukaryotes[30,62] were used as references (cf. halophilic archaea[29, 67]). The lipid analysis provides evidence of the carbonate-hosted ANME-2 community[68], as shown in Supplementary Table S1. We could not compile the raw data set on the main Fig. 5, but it is important to note the $^{13}C$-depleted sugar description[69] of sedimentary ANME-1 and some laboratory-based culture studies that carbon isotopic compositions of amino acids synthesized by photoautotrophs (*e.g.*, *Anabaena* sp.[70] and *Skeletonema costatum*[71]) show similar $^{13}C$-isotopic fractionation patterns for protein amino acids.

**Supplementary Movie S1 is available on this site.**

Methane leakage vent from the anoxic seafloor at the Black Sea:

© Marum & MPI Marine Microbiol. Meteor M72/2 expedition

File: Supplementary MovieS1.mov

a

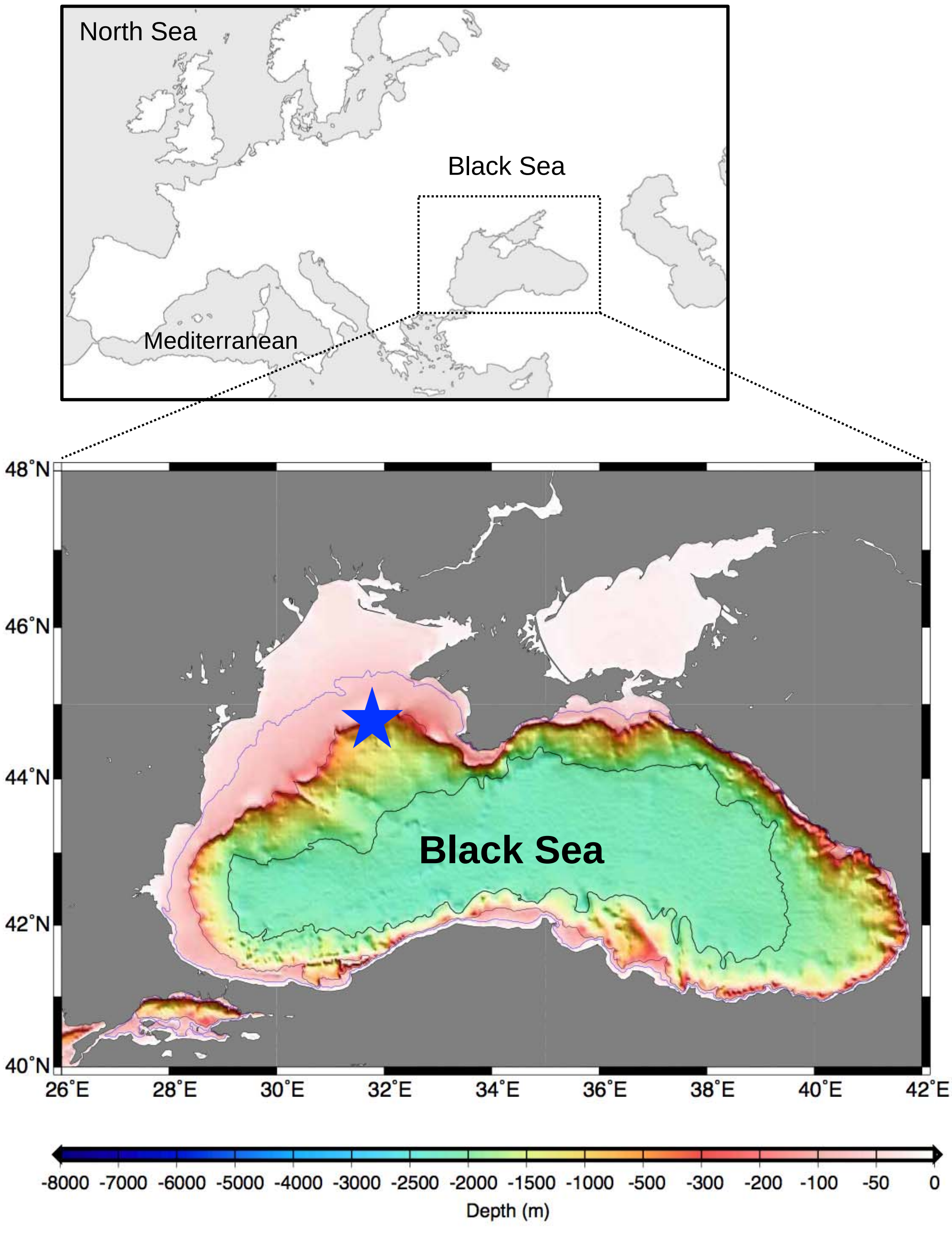


**Supplementary Fig. S1 Sampling location and procedure. (a)** Geological map of the sampling location (star symbol) at the Black Sea. The bathymetry map was modified from ref. [63, 64]. The depth scale is indicated by the colour contour: The contours are for isobaths of 50 m (blue curve) and 2000 m (thick black curve). Please see also other chronological data[64] and water column biomarkers for $\delta^{13}C$ and $\Delta^{14}C$ at the northwestern Black Sea (43°58.88' N, 31°30.83')[65] with the $\Delta^{14}C$ notation[66]. The benthic microbial mats on the methane seep was first described by Ivanov and co-workers[73].

**b**

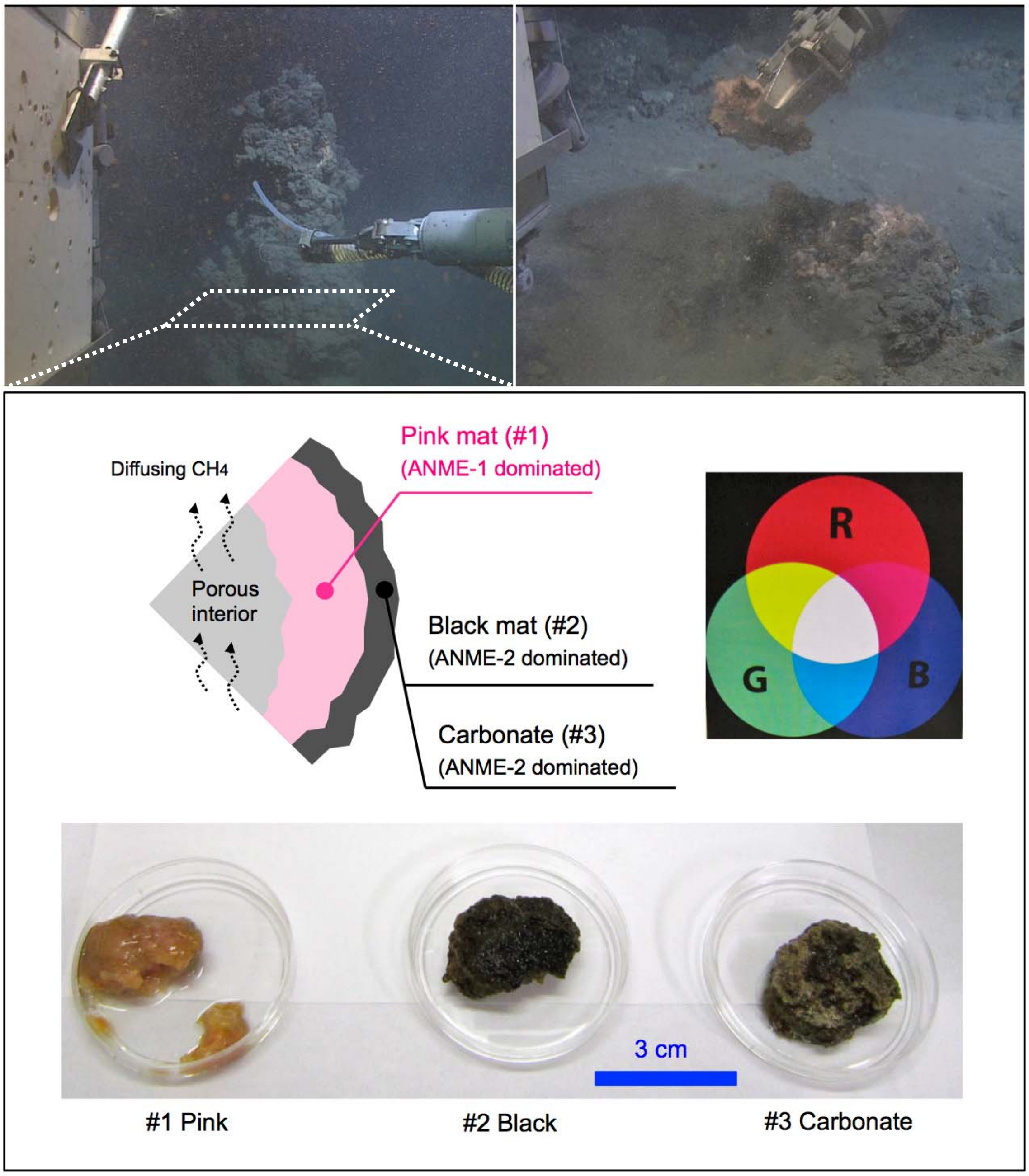


**Supplementary Fig. S1 (b)** Photograph of sampling procedure with robot arm (photo credit: *R/V Meteor* cruise M72/1 science party, ref. 31) and schematic and sample photographs of pink mat (ANME-1 dominated), black mat, and carbonate precipitate (ANME-2 dominated) from the Black Sea shelf (depth: 235 m below sea-level). The photo image was scaled by with the colour chart of red (R), green (G), and blue (B).

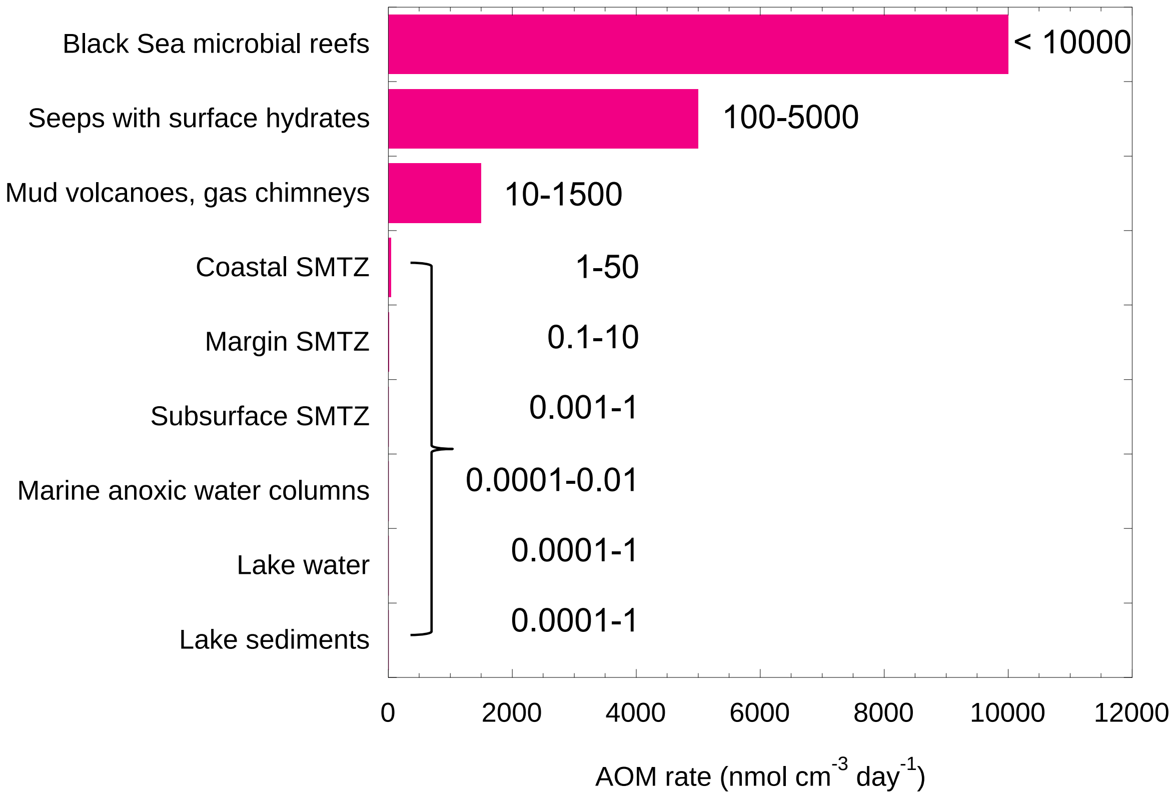


**Supplementary Fig. S2 Global AOM rates in various microbial habitats.** A clear profile of ANME communities on the seep sites of the northwestern Black Sea, with other reference sites including methane hydrates, mud volcanoes, coastal sulphate-methane transition zone (SMTZ), margin SMTZ, marine anoxic water column, lake water and lake sediment. Compilation from refs. [20, 21, 72].

**a**

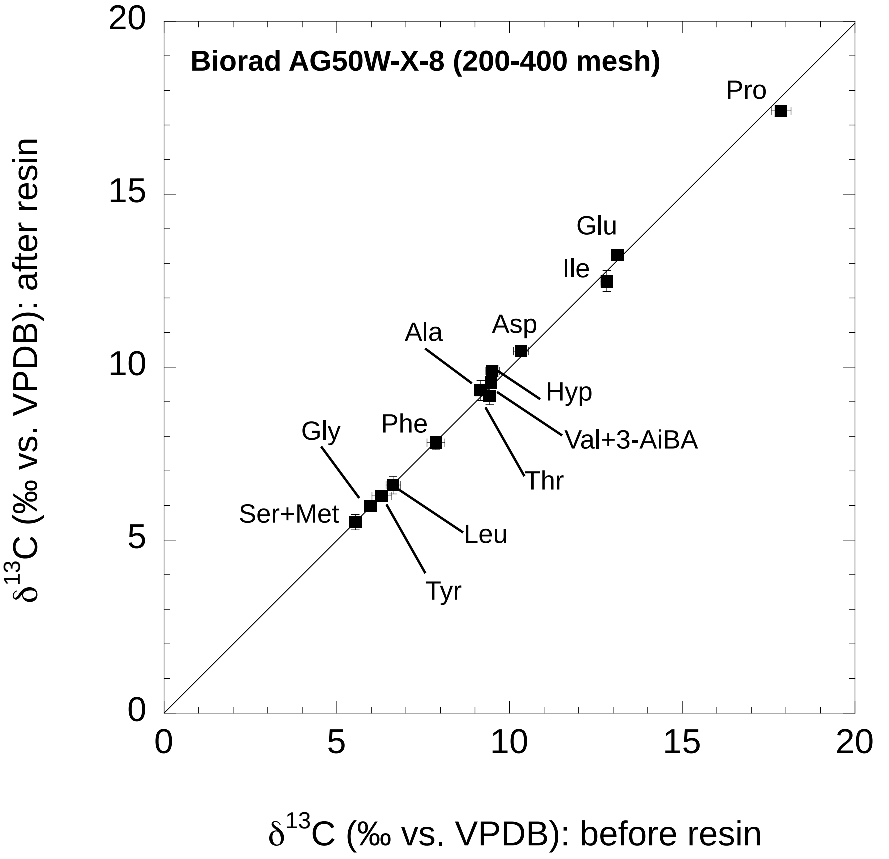


**Supplementary Fig. S3 Analytical accuracy for the treatment of amino acids. (a)** Verification of data consistency before (without) and after cation-exchange chromatography to determine the compound-specific carbon isotopic composition ($\delta^{13}C$, ‰ vs. VPDB) using AG 50W-X8 cation exchange resin (Bio-Rad; 200–400 mesh) with the same chromatographic procedure and amino acid standards[48]. 3-AiBA: 3-aminoisobutyric acid. Abbreviations: Pro, proline; Glu, glutamic acid; Ile, isoleucine; Asp, aspartic acid; Ile, isoleucine; Hyp, hydroxyproline; Val, valine;; Thr, threonine; Ala, alanine; Phe, phenylalanine; Leu, leucine; Tyr, Tyrosine; Gly, glycine; Ser; serine; Met, methionine. The average recovery of a standard amino acid solution was >94% without carbon isotope fractionation during the cation-exchange chromatography. Analytical errors (1σ) of the carbon isotopic composition were better than ± 0.3‰ in both treatments prior to GC separation.

b

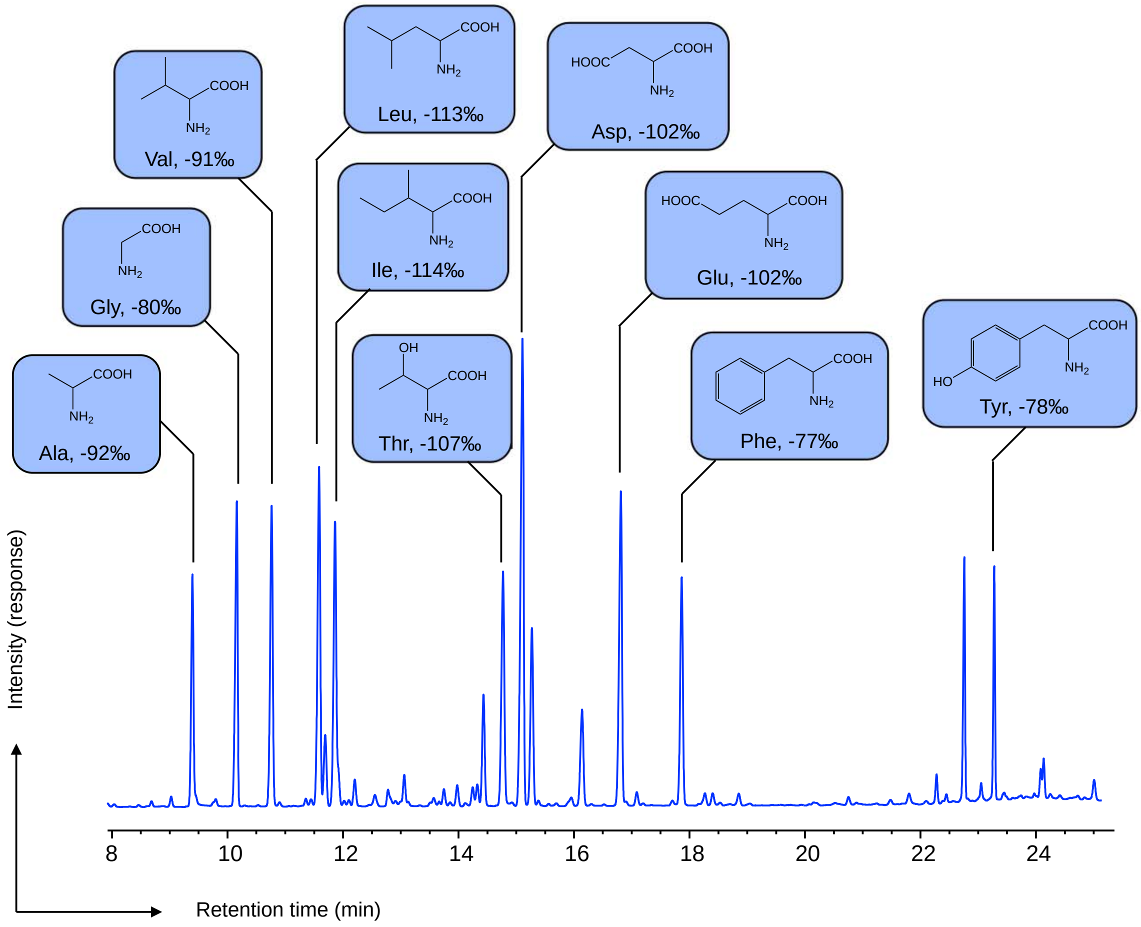


**Supplementary Fig. S3 (b)** Baseline separation of amino acids from the ANME-2 dominated carbonate section analysed by GC/FID with the corresponding carbon isotopic composition (‰, relative to VPDB standard) determined by online GC/C/IRMS (refs. 45-48). Abbreviations: Ala, alanine; Gly, glycine; Val, valine; Leu, leucine; Ile, isoleucine; Thr, threonine; Asp, aspartic acid and asparagine after hydrolysis; Glu, glutamic acid and glutamine after hydrolysis; Phe, phenylalanine; Tyr, tyrosine.

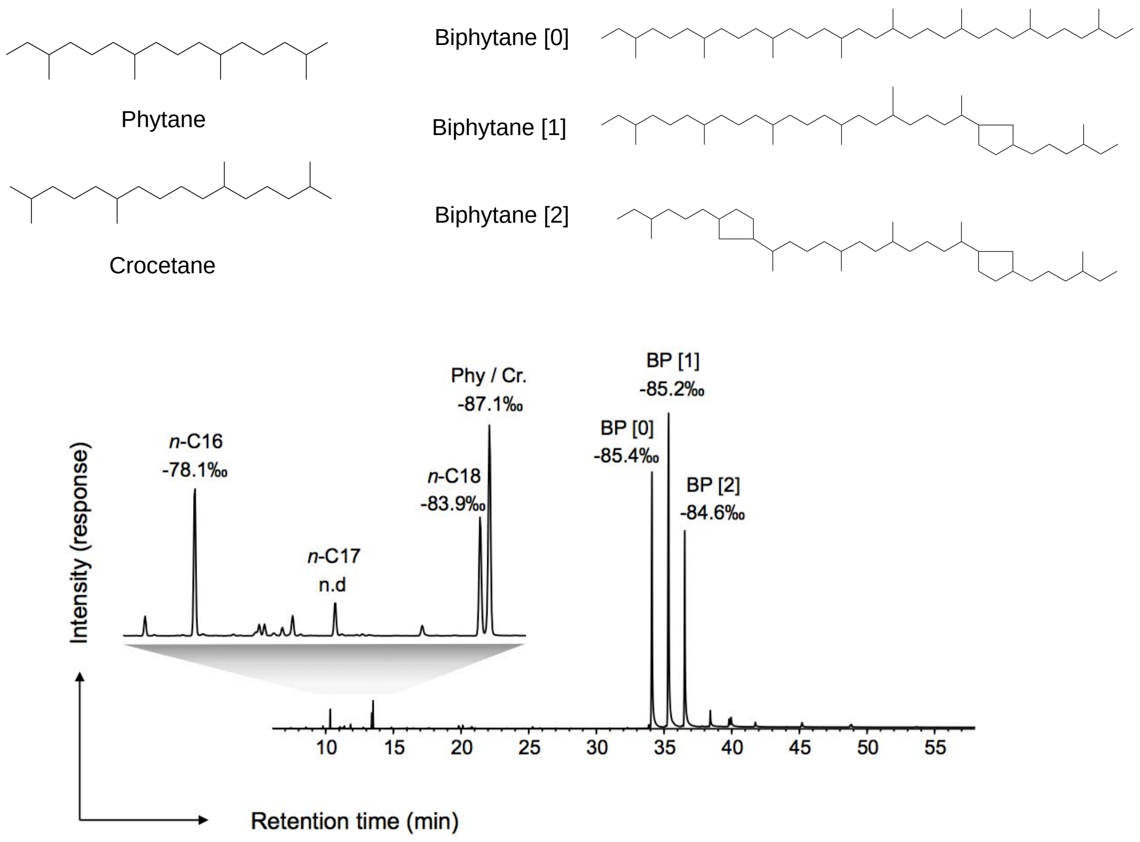


**Supplementary Fig. S4 Evidence of $^{13}$C-depleted lipids from ANME-1.** Baseline separation of archaeal ether-lipids (*i.e.*, $C_{20}$ and $C_{40}$ isoprenoids; phytane/crocetane, biphytanes [0, 1, 2-ring] identified by the authentic standards) and bacterial lipids (*i.e.*, *n*-alkyl lipids) from the ANME-1-dominated pink mat by GC/FID with the corresponding carbon isotopic composition (‰, vs. VPDB) determined by online GC/C/IRMS. The carbon isotopic composition of the ANME-2-dominated black mat and carbonate included the major archaeal $C_{20}$ isoprenoid (< -116‰, vs. VPDB). The lipid analysis provides evidence of the carbonate-hosted ANME-2 community[67]. The chemical structures and names of each compound are shown.

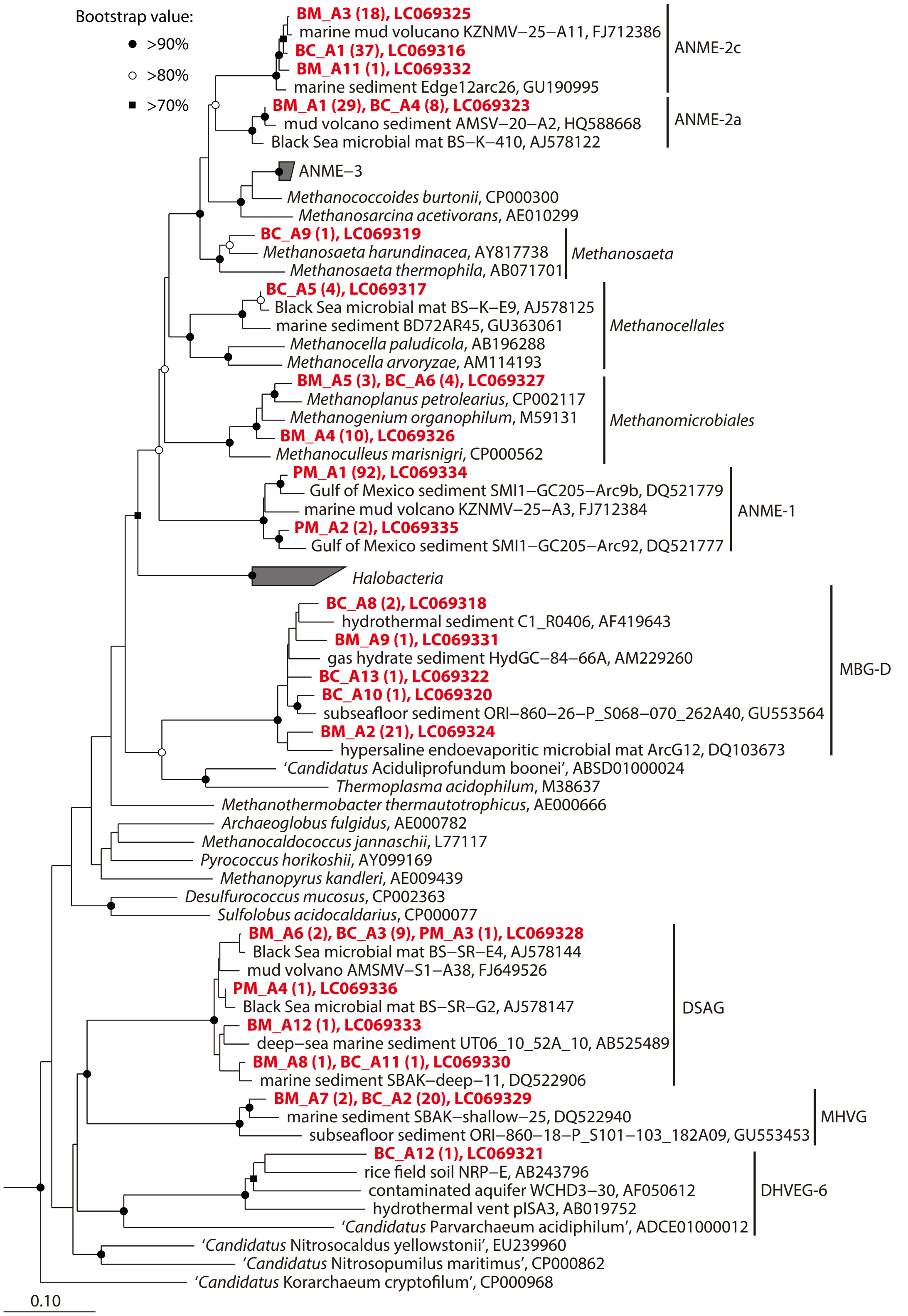


**Supplementary Fig. S5 Phylogenetic tree showing the placement of the 16S rRNA gene clones.** The red coloured phylotypes represent those obtained in the present study.

The name of each phylotype consists of the sample name, an abbreviation of the sample (PM, pink mat; BM, black mat; BC, carbonate), and the phylotype (for example, PM_A1 is phylotype A1 from the pink mat sample). The number in parentheses after the phylotype indicates the number of identical clones. The accession numbers are also shown after each strain or sequence name. The initial tree was constructed with sequences of >1000 nucleotides using the neighbour-joining method. Subsequently, the sequences obtained in the present study were inserted into the tree by using the parsimony insertion tool of the ARB program. Three bacterial 16S rRNA gene sequences, *Bacillus subtilis* (ABQL01000001), *Escherichia coli* (X80725), and *Aquifex pyrophilus* (M83548), were used to root the tree (not shown). The scale bar indicates the estimated number of base changes per nucleotide position. The symbols at the branch nodes show the bootstrap values (>70% indicated only) obtained after 1000 re-samplings.

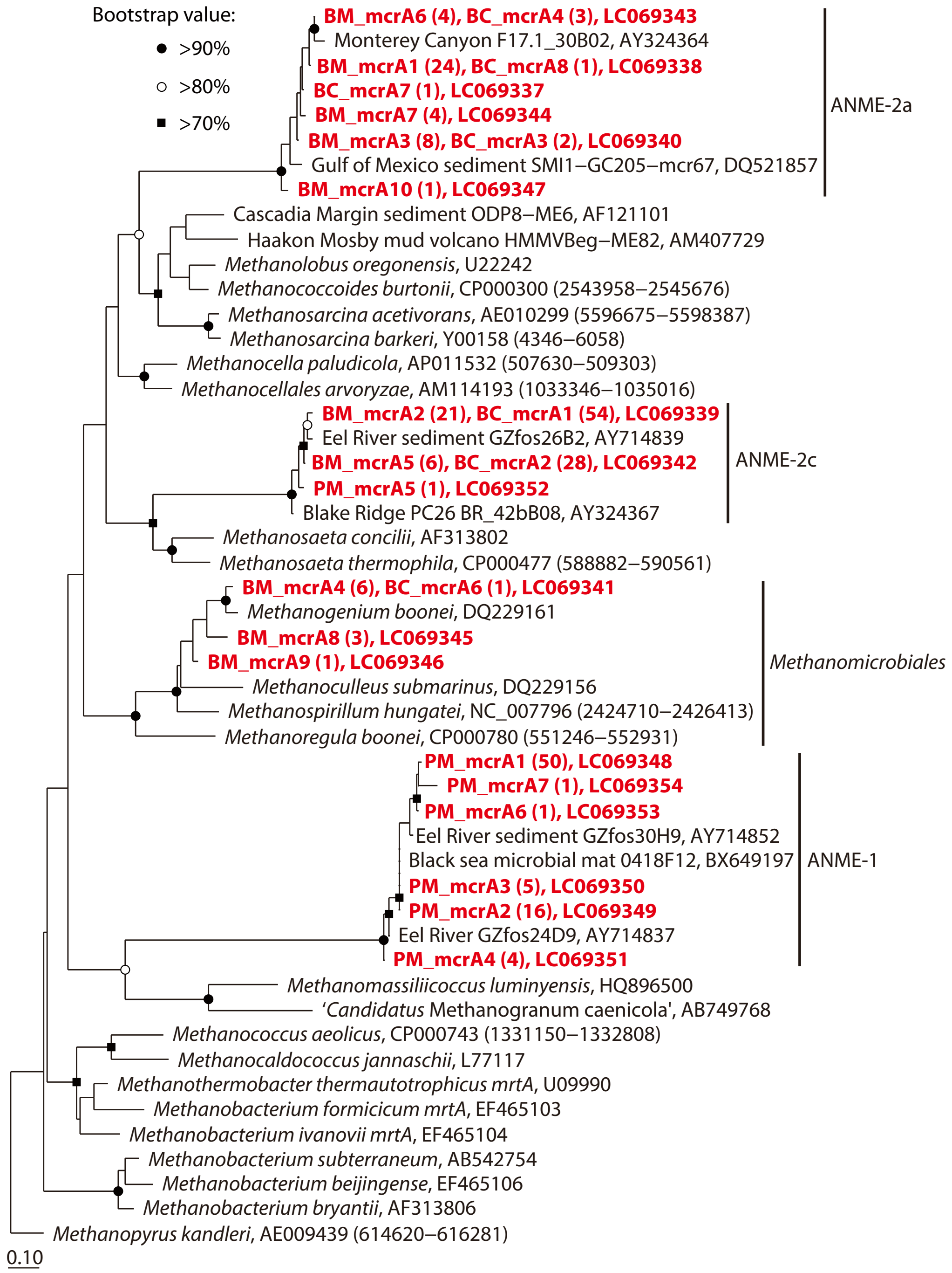


**Supplementary Fig. S6 Phylogenetic tree of the deduced McrA amino acid sequences showing the phylogenetic positions of clones.** The coloured sequences, phylotype names, and numbers in parentheses after the phylotypes are as described in the legend for Fig. S6. The tree was constructed based on distance metrics by using the neighbour-joining method. The sequence of *Methanopyrus kandleri* was used as the outgroup

reference. The scale bar indicates 10% estimated sequence divergence. The accession numbers are shown after each strain or clone name. For sequences taken from complete genomes or the *mcr* operon, the position on the genome/operon is indicated in brackets. The symbols at the branch nodes show the bootstrap values (>70% indicated only) obtained after 1000 re-samplings.

**Supplementary Table S1. Carbon isotopic composition of amino acids and ether lipids extracted from the ANME samples.** Accurate analysis and baseline resolution of threonine, tyrosine, and aspartic acid were achieved using the HP-INNOWAX column, the HP-1 column, and the joint DB-23 and Ultra-2 column, respectively, as noted in the methods section[46,47]. The bulk carbon and nitrogen isotopic composition of ANME-1, ANME-2-dominated mats and the carbonate sample were also shown.

| Amino acids | | | ANME samples ($\delta^{13}C$, ‰ vs. VPDB) | | |
|---|---|---|---|---|---|
| **Name** | **Abb.** | **C*n*** | **Pink (ANME-1 dominated)** | **Black (ANME-2 dominated)** | **Carbonate (ANME-2 dominated)** |
| Glycine | Gly | 2 | -59 | -80 | -93 |
| Alanine | Ala | 3 | -66 | -92 | -98 |
| Aspartic acid | Asp | 4 | -82 | -102 | -104 |
| Threonine | Thr | 4 | -83 | -107 | -105 |
| Glutamic acid | Glu | 5 | -73 | -102 | -98 |
| Valine | Val | 5 | -76 | -91 | -93 |
| Isoleucine | Ile | 6 | -97 | -114 | -109 |
| Leucine | Leu | 6 | -99 | -113 | -108 |
| Phenylalanine | Phe | 9 | -67 | -77 | -77 |
| Tyrosine | Tyr | 9 | n.d. | -78 | -83 |

| Ether lipids | | | ANME samples ($\delta^{13}C$, ‰ vs. VPDB) | | |
|---|---|---|---|---|---|
| **Name** | **Abb.** | **C*n*** | **Pink (ANME-1 dominated)** | **Black (ANME-2 dominated)** | **Carbonate (ANME-2 dominated)** |
| Phytane/ Crocetane | Phy / Cr | 20 | -87.1 | -116.0 | -121.8 |
| Biphytane [0] | BP[0] | 40 | -85.4 | n.d. | n.d. |
| Biphytane [1] | BP[1] | 40 | -85.2 | n.d. | n.d. |
| Biphytane [2] | BP[2] | 40 | -84.6 | n.d. | n.d. |

| | Pink (ANME-1 dominated) | Black (ANME-2 dominated) | Carbonate (ANME-2 dominated) |
|---|---|---|---|
| | | **$\delta^{13}C$, ‰ vs. VPDB** | |
| | -64.1 (*n* = 1) | -79.5 ± 2.9 (*n* = 4) | -44.0 ± 4.0 (*n* = 4) |
| **Bulk analysis** | | **$\delta^{15}N$, ‰ vs. Air** | |
| | -1.0 (*n* = 1) | +1.1 ± 0.2 (*n* = 4) | -0.2 ± 0.5 (*n* = 4) |
| | | **$\Delta^{14}C$, ‰** | |
| | -815.3 ± 1.4 | -770.4 ± 1.8 | -855.9 ± 1.6 |

**References**

54 Miyashita, A. *et al.* Development of 16S rRNA gene-targeted primers for detection of archaeal anaerobic methanotrophs (ANMEs). *FEMS Microbiol. Lett.* **297**, 31-37 (2009).

55 DeLong, E. F. Archaea in coastal marine environments. *PNAS* **89**, 5685-5689 (1992).

56 Luton, P. E., Wayne, J. M., Sharp, R. J. & Riley, P. W. The *mcr*A gene as an alternative to 16S rRNA in the phylogenetic analysis of methanogen populations in landfill. *Microbiol.* **148**, 3521-3530 (2002).

57 Altschul, S. F. *et al.* Gapped BLAST and PSI-BLAST: a new generation of protein database search programs. *Nucleic Acids Res.* **25**, 3389-3402 (1997).

58 Imachi, H. *et al.* Non-sulfate-reducing, syntrophic bacteria affiliated with *Desulfotomaculum* cluster I are widely distributed in methanogenic environments. *Appl. Environ. Microbiol.* **72**, 2080-2091 (2006).

59 Ludwig, W. *et al.* ARB: a software environment for sequence data. *Nucleic Acids Res.* **32**, 1363-1371 (2004).

60 Kanehisa, M. & Goto, S. KEGG: Kyoto encyclopedia of genes and genomes. *Nucleic Acids Res.* **28**, 27-30 (2000).

61 Shieh, J. M. Mesbah, W.B.Whitman, Pseudoauxotrophy of *Methanococcus voltae* for acetate, leucine, and isoleucine. *J. Bacteriol*. **170**, 4091-4096 (1988).

62 Ginger, M.L. *et al.* The biosynthetic incorporation of the intact leucine skeleton into sterol by the trypanosomatid *Leishmania mexicana. J. Biol. Chem.* **276**, 11674-11682 (2001).

63 Liu, K. & Dittert, N. Web-based electronic supplements, Appendix C. In *Carbon and nutrient fluxes in continental margins*. Springer, Berlin. (2010). (http://cmtt.pangaea.de/, on the access in 15-Dec-2013).

64 Jones, G.A. & Gagnon, A.R. Radiocarbon chronology of Black Sea sediments. *Deep Sea Res. Part I*, **41**, 531-557 (1994).

65 Wakeham, S.G. & McNichol, A.P. Transfer of organic carbon through marine water columns to sediments–insights from stable and radiocarbon isotopes of lipid biomarkers. *Biogeosci*. **11**, 6895-6914 (2014).

66 Stuiver, M. & Polach, H. A. Reporting of $^{14}C$ Data - Discussion. *Radiocarbon* **19**, 355-363 (1977).

67 Yamauchi, N. & Tanoue, R. Deuterium incorporation experiments from (3*R*)-and (3*S*)-[3-$^{2}$H] leucine into characteristic isoprenoidal lipid-core of halophilic archaea suggests the involvement of isovaleryl-CoA dehydrogenase. *Biosci. Biotech. Biochem.* **81**, 2062-2070 (2017).

68 Marlow, J. J. *et al.* Carbonate-hosted methanotrophy represents an unrecognized methane sink in the deep sea. *Nature Commun.* **5**, 5095, doi:10.1038/ncomms6094 (2014).

69 Lin, Y.S. *et al.* Intramolecular stable carbon isotopic analysis of archaeal glycosyl tetraether lipids. *Rapid Commun Mass Spectrom.* **24**, 2817-2826 (2010).

70 Macko, S.A., Fogel, M.L., Hare, P.E. & Hoering, T.C. Isotopic fractionation of nitrogen and carbon in the synthesis of amino acids by microorganisms. *Chem. Geol.* **65**, 79-92 (1987).

71 McCarthy, M.D., Benner, R., Lee, C., Hedges, J.I. & Fogel, M.L. Amino acid carbon isotopic fractionation patterns in oceanic dissolved organic matter: an unaltered photoautotrophic source for dissolved organic nitrogen in the ocean? *Marine Chem.* **92**, 123-134 (2004).

72 Knittel, K. & Boetius, A. Anaerobic oxidation of methane: progress with an unknown process. *Annu. Rev. Microbiol.* **63**, 311-334 (2009).

73 Ivanov, M. *et al.* Biogeochemistry of the carbon cycle in the zone of Black Sea methane seeps. *Dokl. Akad. Nauk SSSR*, pp. 1235-1240 (1991). (in Russian)
